# Transformed Shoots of *Dracocephalum forrestii* W.W. Smith from Different Bioreactor Systems as a Rich Source of Natural Phenolic Compounds

**DOI:** 10.3390/molecules25194533

**Published:** 2020-10-03

**Authors:** Izabela Weremczuk-Jeżyna, Paweł Lisiecki, Weronika Gonciarz, Łukasz Kuźma, Magdalena Szemraj, Magdalena Chmiela, Izabela Grzegorczyk-Karolak

**Affiliations:** 1 Department of Biology and Pharmaceutical Botany, Medical University of Lodz, 1 Muszyńskiego Str., 90-001 Lodz, Poland; lukasz.kuzma@umed.lodz.pl (Ł.K.); izabela.grzegorczyk@umed.lodz.pl (I.G.-K.); 2 Department of Pharmaceutical Microbiology and Microbiological Diagnostic, Medical University of Lodz, 137 Pomorska Str., 90-235 Lodz, Poland; pawel.lisiecki@umed.lodz.pl (P.L.); magdalena.szemraj@umed.lodz.pl (M.S.); 3 Department of Immunology and Infectious Biology, Faculty of Biology and Environmental Protection, University of Lodz, 12/16 Banacha Str., 90-237 Lodz, Poland; weronika.gonciarz@biol.uni.lodz.pl (W.G.); magdalena.chmiela@biol.uni.lodz.pl (M.C.)

**Keywords:** acacetin, plant bioreactor, *Dracocephalum forrestii*, transformed shoots, rosmarinic acid

## Abstract

Transformed shoots of the Tibetan medicinal plant *Dracocephalum forrestii* were cultured in temporary immersion bioreactors (RITA and Plantform) and in nutrient sprinkle bioreactor (NSB) for 3 weeks in MS (Murashige and Skoog) liquid medium with 0.5 mg/L BPA (*N*-benzyl-9-(2-tetrahydropyranyl)-adenine) and 0.2 mg/L IAA (indole-3-acetic acid). The greatest biomass growth index (GI = 52.06 fresh weight (FW) and 55.67 dry weight (DW)) was observed for shoots in the RITA bioreactor, while the highest multiplication rate was found in the NSB (838 shoots per bioreactor). The levels of three phenolic acids and five flavonoid derivatives in the shoot hydromethanolic extract were evaluated using UHPLC (ultra-high performance liquid chromatography). The predominant metabolite was rosmarinic acid (RA)—the highest RA level (18.35 mg/g DW) and total evaluated phenol content (24.15 mg/g DW) were observed in shoots grown in NSB. The NSB culture, i.e., the most productive one, was evaluated for its antioxidant activity on the basis of reduction of ferric ions (ferric reducing antioxidant power, FRAP) and two scavenging radical (O_2_*^•^*^–^ and DPPH, 1,1-diphenyl-2-picrylhydrazyl radical) assays; its antibacterial, antifungal, and antiproliative potential against L929 cells was also tested (3-[4,5-dimethylthiazole-2-yl]-2,5-diphenyltetrazolium bromide (MTT) test). The plant material revealed moderate antioxidant and antimicrobial activities and demonstrated high safety in the MTT test—no cytotoxicity at concentrations up to 50 mg/mL was found, and less than a 20% decrease in L929 cell viability was observed at this concentration.

## 1. Introduction

Plant biotechnology allows great potential for obtaining rare plant material for use in agriculture and the pharmaceutical industry. However, conventional tissue culture techniques are limited by their small scale and high cost. A potential alternative for obtaining valuable medicinal plants and their metabolites for commercial aims is the use of plant bioreactors. Many bioreactor systems have been developed in the past few years [1]. Among them, the two most promising approaches for the scaling-up of in vitro plant propagation are the temporary immersion bioreactor system (TBIS) and nutrient sprinkle bioreactor (NBS). In TBIS, the plant cultures are immersed in the medium in a specific period of time at specified intervals, while in NSB, the cultures are supported on a porous base and are periodically sprayed with medium. These bioreactor systems are beneficial for plant cultures because they are not exposed to continuous immersion or shear force, as observed in mechanically agitated or air-driven submerged bioreactors [2]. Additionally, TBIS and NSB are more effective for in vitro plant cultivation compared to culture plant on semi-solid or solid media [3,4].

Both bioreactor systems offer increased culture multiplication and high biomass accumulation due to high nutrient availability. Compared to traditional cultivation in a liquid medium, bioreactors offer reduced hyperhydricity and physiological stress [5], as well as lower medium costs (i.e., elimination of gelling agent) and shorter plant propagation time. They enable easier handling and reduce labor coast [6,7,8]. Several crop and medicinal plants have been cultured in NSB and TIBS thus far, for example *Ananas comosus* [9], *Solanum tuberosum* [10], *Digitalis lanata* [11], and *Rehmania glutinosa* [12]. Plant breeding in bioreactor systems could also be a simple method for optimizing secondary metabolite production in various plant organs. Reports describe the biosynthesis of caredenolides in bioreactor-cultivated shoots of *D. lanata* [11], sequiterpene lactones in shoots of *Thapsia garganica* [13], and phenolic compounds in shoots of *Scutellaria alpina* [14] and *Schisandra chinensis* [15].

*Dracocephalum forrestii* W.W. Smith (Lamiaceae) is a Tibetan medical plant that is native to the mountain regions of Yunnan province (China) [16]. In traditional Chinese medicine, aerial parts of *D. forrestii* are used as diuretic, astringent, and antipyretic agents [17,18]. These properties are conditioned by the secondary metabolites present in the plant, with the flavonoids, lignans, terpenoids, and phenolic acids being most frequently mentioned in the literature [17,18]. It is known that *Dracocephalum* species plants are a particularly good source of phenolic compounds such as the following caffeic acid derivatives: rosmarinic acid; chlorogenic acid; and salvianolic acid B and flavonoid glycosides, e.g., acacetin and apigenin derivatives [17,18,19,20,21]. 

Weremczuk-Jeżyna et al. [22] described the obtaining and metabolite profile of *D. forrestii* transformed roots, on which the shoots regenerated spontaneously. The phytochemical analysis of the hydromethanolic extract of the obtained transformed shoots found high production of rosmarinic acid (RA), i.e., its predominant phenolic acid and flavonoid derivative (mainly acacetin glucosides). Acacetin glycosides, identified for the first time in transformed shoots cultured on solid agar media, show antioxidant, anti-inflammatory, and anticancer properties [19,23]. Acacetin derivatives induced apoptosis in a human prostate cancer cell line [24]. A study of the effect of different purine-type cytokinins on the production of phenolic compounds in transformed shoots of *D. forrestii* found the highest amount of RA and acacetin derivatives to accumulate on media with N-benzyl-9-(2-tetrahydropyranyl)-adenine (BPA) [22]. 

The aim of the current study was to evaluate the potential of the scaling-up production of phenolic compounds by transformed *D. forrestii* shoots. Initially, the shoots were cultured on a small scale (in tubes) on a solid medium [22]. Here, for the first time, this culture of *D. forrestti* was carried out in bioreactor conditions. We investigated the effects of three bioreactor systems: two temporary immersion bioreactors (RITA and Plantform) and a nutrient sprinkle bioreactor (NSB). We determined shoot growth and secondary metabolite production in the cultures after 3 weeks, and evaluated the antioxidant, antimicrobial, and cytotoxicity potential of the hydromethanolic extract from shoots grown in the NSB, with this culture being characterized by the highest production of phenolic compounds.

## 2. Results and Discussion

### 2.1. Culture Growth

The growth and development of plant culture in bioreactor conditions is mainly dependent on the supply of oxygen and nutrients, which is governed by the circulation, mixing, and aeration of the liquid medium [25]. In our study, the transformed shoot of *D. forrestii* were cultured on a large scale in three types of bioreactors. Two of these, Rita and Plantform, are temporary immersion bioreactors, in which the explants were not immersed permanently in liquid medium—after a short immersion period, the medium returned to a reservoir by gravity. The two systems differ from one another in terms of flow rate and the pressure at which the liquid medium is supplied to the plant culture. The third approach employed a bioreactor (NSB) system, in which liquid medium was sprinkled at specified intervals.

The explants (shoots with three or four nodal segments) were incubated in MS (Murashige and Skoog) liquid medium with the addition of 0.5 mg/L BPA and 0.2 mg/L indole-3-acetic acid (IAA). In all types of tested bioreactors, the transformed shoots of *D. forrestii* showed intensive growth after 3 weeks (Table 1). The highest multiplication rate, i.e., the number or shoots/buds per explant, was found to be about 70, and this was observed in the NSB; in contrast, these values were 47 for RITA and 17 for Plantform (Table 1). In all cases, the number of obtained shoots was significantly higher than those observed for transformed shoots cultivated on MS agar medium with the same growth regulators (7.2 shoots per explant) [22]. Moreover, cultivation in bioreactors was faster; this fact, together with the elimination of agar from the medium, significantly reduces the cost of cultivation.

The greatest transformed shoot growth was observed in the NSB; this may have been due to the plant tissue being given uninterrupted access to the gas phase [26]. The NSB system has been used successfully for the propagation of medicinal plants such *S. alpina* [14] and *S. chinensis* [27], as well as non-transformed *D. forrestii* shoots [28]. However, the temporary immersion bioreactors have proven effective for the propagation of different medicinal plants such as *Betula pubescens*, *Coffea conephora*, or *Stevia rebaudiana* [4,29,30].

Some of the transformed *D. forrestii* shoots in all used bioreactors demonstrated hyperhydricity (glassy and transparent shoots with brittle leaves). The highest number of hyperhydric shoots were found in NSB (24%), and the lowest in the Plantform system (5%) (Table 1). Hyperhydricity has been described for several plant species grown in liquid media [3]. According to González et al. [31], the number of vitreous shoots increased with excessive hydration, which in turn depended on the time and/or frequency of immersion. However, immersion could not have been the cause of hyperhydricity in our study, as a similar or lower percentage of hyperhydric *D. forrestii* shoots were observed in all bioreactor systems compared with that of agar medium (25%) [22].

**Figure 1:**
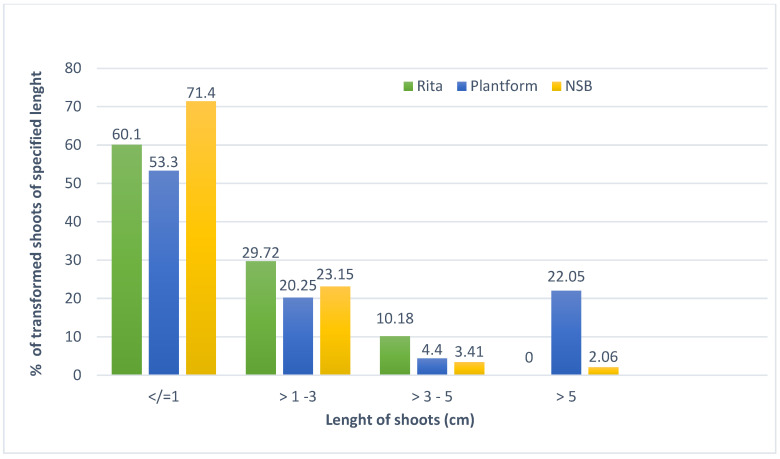
The length of *D. forrestii* transformed shoots after 3 weeks in different bioreactor systems in MS medium with BPA 0.5 mg/L and IAA0.2 mg/L.

The obtained transformed *D. forrestii* shoots were of similar length, regardless of the type of bioreactor. Most shoots did not exceed 1 cm in length (Figure 1). This effect could be due to elimination of apical dominance and is often observed in in vitro cultures grown in the presence of cytokinin in the medium [32].

**Figure 2:**
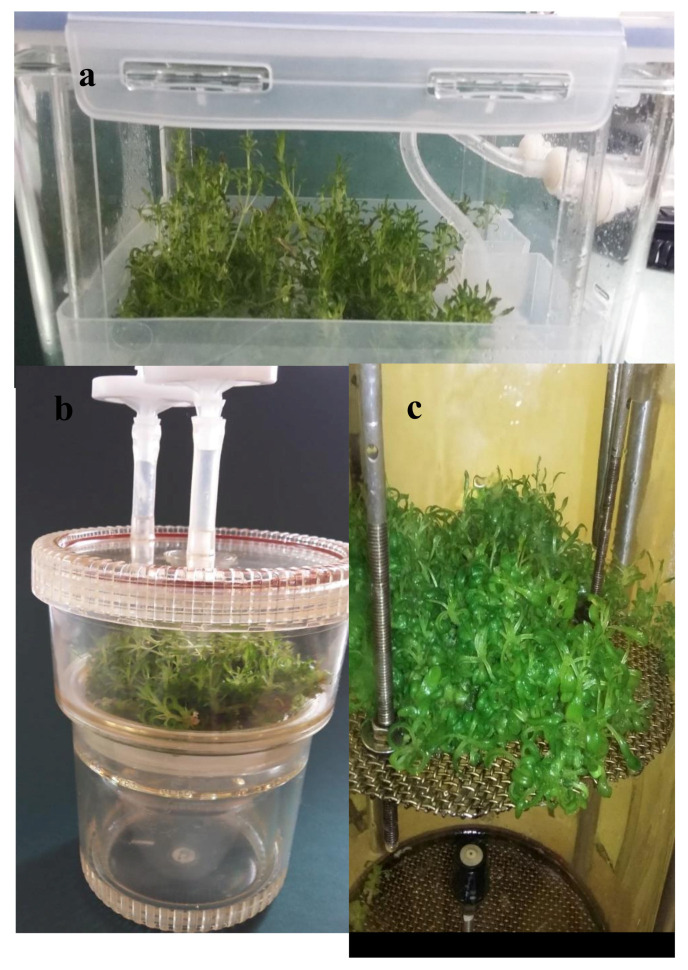
The *D. forrestii* transformed shoots grown for 3 weeks in MS medium with BPA 0.5 mg/mL and IAA 0.2 mg/mL in Plantform bioreactor (**a**), RITA (**b**) bioreactor, and nutrient sprinkle bioreactor (**c**).

Bioreactor systems enabled efficient growth of *D. forrestii* transformed shoot culture (Figure 2). The highest biomass after 3 weeks was observed in the RITA bioreactor (growth index (GI): 52.06 for fresh weight (FW) and 55.67 for dry weight (DW)) followed by the NSB system (43.48 FW and 44.55 DW). Significantly higher biomass was obtained compared to shoots cultivated on solid medium for 4 weeks (24.5 and 27.33, respectively) [22]. Similar differences have been observed for other plant species. Moreover, the NSB and RITA bioreactors have been found to be effective bioreactor systems for *Schisandra chinensis* microshoot culture growth [15]. This high biomass accumulation could be caused by better absorption of nutrients and hormones from the liquid medium [26,33]. However, lower growth parameters were observed for shoots grown in Plantform bioreactor (Table 1). In our study, this stunted growth may result from the lower availability of oxygen to the shoots while being flooded in the Plantform bioreactor—this parameter turned out to be a limiting factor for large-scale cultivation of *Dracocephalum* shoots.

### 2.2. Phytochemical Analysis

The hydromethanolic extracts from large-scale transformed shoots of *D. forrestii* were evaluated for the production of phenolic compounds. UPLC–PDA–ESI–MS (ultra performance liquid chromatography-electrospray-ionization-mass spectrometry) analysis found eight polyphenolic compounds also identified in shoots grown on solid medium: three phenolic acids (chlorogenic acid, dicaffeoylquinic acid, rosmarinic acid) and five flavonoid glucosides (acacetinrhamnosyl-trihexoside, acacetin acetyl-rhamnosyl-trihexoside, apigenin caffeoyl-rhamnoside, apigenin p-coumaroyl-rhamnoside I, apigenin p-coumaroyl-rhamnoside II). Their identification is described in detail by Weremczuk-Jeżyna et al. [22]. The identified phenolic acids are specific for the Lamiaceae family and *Dracocephalum* genus [34,35]. 

The main phenolic acid in the hydromethanolic extract was rosmarinic acid (RA), and all tested bioreactors were found to be good sources of the compound. The highest RA (18.35 mg/g DW) content was found in the NSB (Table 2). Similar levels of RA, 17.9 mg/g DW, were found in non-transformed shoots of *D. forrestii* cultures in an NSB [28] and in the transformed shoot culture grown on agar (0.7%) MS medium with the same combination of growth regulators (16.9 mg/g DW); however, this required a longer period of shoot growth, i.e., 4 weeks [22]. The accessibility of nutrients from the liquid medium and easy gas exchange influence not only the growth of the cultures, but also their secondary metabolism [36]. Therefore, plant tissue culture in bioreactor can produce higher amounts of compounds than in vitro culture grown on solid medium. Weremczuk-Jeżyna et al. [22] report that non-transformed shoots of *D. forrestii* cultivated in NSB produced higher amounts of salvianolic acid B than shoots grown on agar, and Grzegorczyk and Wysokińska [37] note a higher concentration of carnosic acid in *Salvia officinalis* shoots grown in NSB than in shoots cultured in static liquid medium.

The *D. forrestii* shoots cultivated in the RITA and Plantform systems produced lower amounts of RA: 11.91 mg/g DW and 8.23 mg/g DW, respectively (Table 2). However, these RA levels were several times higher than in the aerial parts of one-year-old intact plants (3.81 mg/g DW) [38]. The therapeutic potential of RA is well known [39]. RA is believed to be the strongest antioxidant of all hydroxycinnamic acid derivatives [40]; it reduces ROS and lipid peroxidation, and has demonstrated antibacterial, antidepressant, anti-aging, nephroprotective, and hepatoprotective properties [41,42]. It also affects tumor cell proliferation, necrosis, and apoptosis [43].

The other phenolic acids identified in *D. forrestii* transformed shoots also have a wide spectrum of biological activity. Chlorogenic acid has been proven to have antioxidant, anti-inflammatory, and anti-carcinogenic properties [44,45,46] and influences lipid and glucose metabolism [47,48]. Similarly, the dicaffeoylquinic acids demonstrate antioxidant and antiviral activity, particularly against HIV integrase and HIV replication in tissue culture [49,50,51,52]. Chlorogenic acid and dicaffeoylquinic acid were previously detected in *D. forrestii* non-transformed shoots grown in an NSB; however, chlorogenic acid production was less than 5% of that found in transformed shoots grown in all tested bioreactors, and dicaffeoylquinic acid level was only present in low quality, as noted previously [28]. The compounds in transformed shoot cultured in bioreactors and on solid media were present in similar amounts [22]. 

The acacetin glycosides, particularly their monoglycosides, have already been identified in aerial parts of other *Dracocephalum* species, for *Dracocephalum ruyschiana* or *Dracocephalum foetidum* [19,20] and also in transformed shoots of *D. forrestii* grown on agar-solidified media [22]. Several studies have reported that acacetin glycoside derivatives inhibited enzymes such as acetylcholinesterase and hyaluronidase [20,53]. In addition, these compounds are expected to have antioxidant, anti-inflammatory, and anti-cancer activity [20,54,55]. 

The transformed *D. forrestii* shoot culture in the three described bioreactor systems proved to be effectively source of acacetin glucosides. The transformed shoots grown in the three bioreactor types demonstrated similar levels of acacetin rhamnosyl-trihexoside (0.91–1.01 mg/g DW) and acacetin acetylrhamnosyl-trihexoside (2.25–2.45 mg/g DW) (Table 2). These levels were two- and threefold lower than these obtained in transformed shoots grown on solid medium [22]. 

Other flavonoids found in the shoot extract were apigenin derivatives: two apigenin *p*-coumaroyl-rhamnoside and apigenin caffeoyl-rhamnoside. Although simple apigenin derivatives are commonly found in plants, the above or similar compounds are rarely detected in plant material, and little is known of their potential properties. The apigenin p-coumaryl-glycosides isolated from *Clematis tangulica* demonstrated antioxidant capacity and a neuroprotective effect, which was induced by STAT3 (signal transducer and activator of transcription) phosphorylation, mediated Mn-SOD (manganes-superoxide dismutase) upregulation [56]. However, Manivannan [57] reports that apigenin caffeolyl-glucoside has anti-inflammatory activity and accelerates wound healing in diseases.

Low levels of apigenin caffeoyl-rhamnoside and apigenin *p*-coumaroyl-rhamnoside I were recorded in *D. forrestii* shoots that were grown in bioreactor systems (Table 2). However, the conditions were effective for the production of the apigenin p-coumaroyl-rhamnoside II (compound 8), whose concentration ranged from 1.76 mg/g DW (NSB) to 2.28 mg/g DW (Plantform bioreactor). The apigenin *p*-coumaroyl-rhamnoside was found to be present in twofold higher amounts than in transformed shoots cultured on MS agar medium with the same plant growth regulators [22]. 

To summarize, the highest productivity of total phenolic compounds (239.5 mg/L medium, 65% of which was RA) was observed in shoots cultivated in the RITA bioreactor—this resulted from the lowest demand for medium (250 mL) in this system during a single growth cycle. Meanwhile, the highest content of compounds expressed as DW was found in the culture maintained in NSB. Etienne and Berthouly [29] propose that the most important parameters influencing the efficiency of bioreactor system are the methods of distribution medium and absorption of nutrients by plant tissue. The high biomass and metabolite production also depend on oxygen supply and plant sensitivity [32]. The NSB demonstrated efficient production of phenylethanoid and flavonoids in shoot culture of *Scutelaria alpina* [14] or flavonoids in microshoot cultures of *Schisandra chinensis* [15]. The RITA bioreactor system was suitable for accumulating sesquiterpene lactones in *Thapsia garganica* shoots [13]. The Plantform bioreactor demonstrated efficient production of lignans in *S. chinnensis* shoots [27].

### 2.3. Antioxidant Potential

The antioxidant properties of extracts from *Dracocephalum* species are mainly associated with the presence of phenolic compounds [55,58,59,60]. The redox activity of these compounds allows them to act as reducing agents, singlet oxygen quenchers, and hydrogen donors [21].

The present study is the first to determine the antioxidant potential of *D. forrestii* transformed shoots grown in an NSB. The hydromethanolic extract was evaluated by three different antioxidant tests: ferric reducing antioxidant power (FRAP), 1,1-diphenyl-2-picrylhydrazyl (DPPH), and NBT (nitroblue terazolinum) radical scavenging assay (Table 3). The studied plant material showed significant antioxidant activity in all used assays. The antiradical properties of the transformed shoots, expressed as EC_50_, was 74.9 μg/mL for DPPH test and 116.1 μg/mL for NBT test. Slightly higher EC_50_ values have previously been found in non-transformed *D. forrestii* shoots grown in the same bioreactor: 30.7 μg/mL and 69.9 μg/mL, respectively [37]. In addition, the FRAP results for transformed shoots of *D. forrestii* were 724.9 μM Fe(II)/g DW, half those of the non-transformed shoots, i.e., 1319 μM Fe(II)/g DW of extract [28]. 

The transformed and non-transformed *D. forrestii* shoots demonstrated different levels of antioxidant activity, despite the similar amounts of total phenolic compounds and RA. This may be due to the presence of salvianolic acid B present in the non-transformed shoots but not the transformed shoots; the compound is known for its antioxidant properties [61] and can support the antioxidant potential of RA. Meanwhile, flavonoids with sucrose and coumaryl groups identified in transformed culture may be less active in this area. It is known that the glycosylation of flavonoid aglycone at the -OH and/or -OCH_3_ groups reduces the free radical scavenging potential [62,63]. Especially complex substituents such as those with multi-sugar may hinder access to the active center of the molecule and limit its action [62].

### 2.4. Antimicrobial Potential

Due to the constantly increasing resistance of pathogenic microorganisms, the search of sources of new agents with antimicrobial potential among plant-based compounds continues [64]. Earlier studies primarily concerned the antimicrobial activity of essential oils obtained from *Dracocephalum* plants [65,66]. A study of the antimicrobial activity of hydromethanolic extract from non-transformed shoots of *D. forrestii* cultured in NSB [28] gave promising results. Hence, the present study examined the antimicrobial properties of hydromehanolic extract from transformed shoot culture was examined against eight human pathogenic bacteria strains (*Staphylococcus aureus*, *Staphylococcus epidermidis*, *Enterococcus faecalis*, *Enterococcus faecium*, *Bacillus cereus*, *Escherichia coli*, *Pseudomonas aeruginosa*, *Proteus vulgaris*) and four human pathogenic fungi (*Candida albicans* ATTC 10,231 and ZMF 1, *Candida* *glabrata*, *Aspergillus brasiliensis*) (Table 4).

The strongest activity and lowest values of minimum inhibitory concentration (MIC) and minimum bactericidal concentration (MBC) (10 mg/mL) were found against Gram-positive bacteria strains *Staphylococcus epidermidis* and *Bacillus cereus*, and Gram-negative bacteria *Escherichia coli* (Table 4). The non-transformed shoots of *D. forrestii* have been found to demonstrate slightly higher antibacterial potential against these bacteria [28]. However, even higher MIC values have been obtained from alcohol extracts of the aerial parts of other *Dracocephalum* species: *Dracocephalum moldavica* extract inhibited growth *E. coli* and *Klebsiella pneumoniae* in the range 10–40 mg/mL [67], and the MIC of the extract from *Dracocephalum kotschyi* against *E. coli* was 25–100 mg/mL. On the other hand, the highest antibacterial activities were seen for ethyl acetate extracts of *D. kotschyi* against *Staphylococcus aureus* (0.78–3.13 mg/mL) and *D. moldavica* against *Bacillus cereus* (0.39–1.56 mg/mL) [68,69]. Other tested bacterial strains were less sensitive to the hydromethanolic extracts of transformed *D. forrestii* culture (Table 4).

The hydromethanolic extract of transformed shoots of *D. forrestii* revealed moderate fungicidal activity against *Aspergillus brasilensis* and three strains of *Candida* (Table 4). The antifungal effect of alcoholic extract of *Dracocephalum* plants has been minimally analyzed. Salman et al. [70] reported that the essential oil of *D. moldavica* displays strong antifungal properties against *Candida albicans* (MIC < 1.56 μm/mL). Weremczuk-Jeżyna et al. [28] report that non-transformed shoots of *D. forrestii* showed better antifungal effect against *Candida* strains (MIC = 2.5 mg/mL). This lower antimicrobial potential of transformed shoots may have been a result of the absence of salvianolic acid B in the extract, as was observed for antioxidant activity [28].

### 2.5. Cytotoxicity Potential

**Figure 3:**
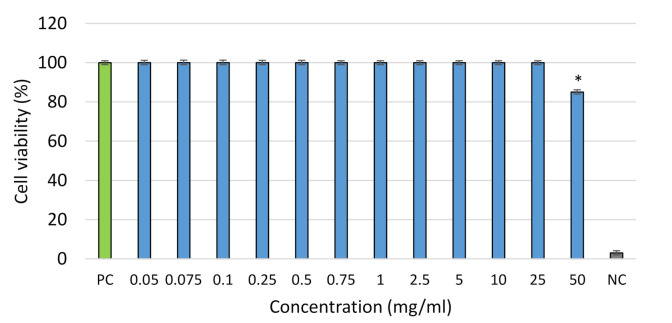
Cytotoxic effect of hydromethanolic extract from transformed shoots of *D. forrestii* grown for 3 weeks in NBS in MS medium with BPA 0.5 mg/L and IAA 0.2 mg/L against L929 cells (blue column). The results are expressed as means of three replicates ± SE. The value followed marked by (*) was significantly different with comparison other according to the Mann–Whitney *U* test (*p* < 0.05). PC: positive control (cells culture in the culture medium alone) (green column), NC: negative control (cells treated with 0.03% H_2_O_2_).

In this work, the hydromethanolic extract of *D. forrestii* transformed shoot was tested on L929 mouse fibroblasts by the 3-[4,5-dimethylthiazole-2-yl]-2,5-diphenyltetrazolium bromide (MTT) assay. The MTT test is based on the reduction of the soluble yellow MTT tertrazolinum salt to a blue insoluble MTT formazan product by mitochondrial succinic dehydrogenase [71]. The effects are given in Figure 3. This is the first report on the subject of *D. forrestii* extract cytotoxic activity. Our findings indicated that the hydromethanolic extract is very safe for mammalian cells and did not cause cell damage at a concentration of 25 mg/mL. The cell viability of L929 only fell below 85% of control values at a concentration of 50 mg/mL (Figure 3). 

No literature data exist on the toxic effect of *Dracocephalum* extracts on normal cell lines; however, some species have demonstrated cytotoxicity activity against cancer lines. For example, Sani et al. [72] reported that alcoholic extract from *D. kotschyi* aerial parts displayed significant cytotoxic activities against lung cancer Calu6 and Melhr-80 cells. In the future, it would be reasonable to check the cytotoxicity of the analyzed extract from transformed shoots of *D. forrestii* against cancer cells.

## 3. Materials and Methods

### 3.1. Transformed Shoot Culture 

The *D. forrestii* transformed shoots were spontaneously regenerated on a hairy root culture grown in WP (Woody Plant) [73] liquid medium in darkness [74]. The procedure of obtaining shoots and optimizing the medium for their growth and secondary metabolite productions is described detail by Weremczuk-Jeżyna et al. [22]. The transformed shoots were cultivated on MS [75] agar (0.7%) medium with the addition of 0.5 mg/L BPA and 0.2 mg/L IAA [22]. Shoot fragments about 4 cm in length with 3–4 nodal segments were used as explants.

### 3.2. Transformed Shoot Culture in Bioreactor Systems

The transformed shoots of *D. forrestii* were cultivated in three types of bioreactors (Figure S1). Two are commercially available temporary immersion systems: Plantform (PlantForm AB, Sweden; Figure 1a) and RITA (Récipient à Immersion Temporiaire Automatiqe, VITROPIC, France; Figure 1b). The Plantform bioreactor, containing 500 mL of liquid growth medium, was supplied with air via Optima pumps (Hagen, Canada) with an inlet capacity of 0.6 m^3^/h. In the RITA bioreactor, containing 250 mL of nutrient medium, the dosage of the liquid medium was controlled using a DT4.4 pressure pump (Becker, Germany) with a capacity of 4.2 m^3^/h and pressure of 1000 mbar. In both temporary immersion bioreactors, the medium was supplied to the shoot culture for 10 min every 80 min. 

The 10 L nutrient sprinkle bioreactor (NSB) contained 1000 mL liquid medium (Figure 1c). The medium dosage was controlled using a CH-8604 peristaltic pump (Chemap AG, Switzerland). The operating time of the pump was 25s every 2.5 min (Chemap AG, Valketswill, Switzerland). Constant medium temperature was maintained with the thermomix MM Thermostat (Sortarius BBJ Systems GmbH, Germany). The NSB was described in detail by Piątczak et al. [12]. The shoots in all types of bioreactors were cultured for 3 weeks.

In all bioreactor systems, the cultures were grown on MS medium with saccharose (30 g/L) and growth regulators: 0.5 mg/L BPA and 0.2 mg/L IAA [19]. In the study, shoots from 16–18 passages were used. The biomass of inoculum was around 0.5 g of fresh weight (FW) (0.06 g of dry weight (DW) for RITA and 0.8 g FW (0.09 g DW) for Plantform and nutrient sprinkle bioreactor. All cultures were kept at 26 ± 2 °C under a 16 h photoperiod provided by cool white fluorescent lamps (40 μmm^−2^s^−1^). In all tested bioreactor systems, we determined the following after 3 weeks of culture: the number shoots per explant, number of shoots per bioreactor and shoot length, as well as FW and DW of culture (g/bioreactor) and growth index (GI) calculated as (final biomass – initial biomass)/initial biomass [37]. Shoot morphology was also recorded.

### 3.3. Phytochemical Analysis

After harvesting, the plant material from all tested bioreactors were frozen (24 h, −20 °C) and lyophilized in condenser temperature −52 °C (Freeze Drying System Alpha 1–2 LD, CHRIST, Germany). Then, the dry samples were powdered and used for phytochemical investigations (100 mg DW). The extraction was carried out using a 8:2 *v*/*v* solution of methanol/water according to Weremczuk-Jeżyna et al. [38]. To determine the phenolic compounds content, we subjected the obtained hydromethanolic extract to ultra-high performance liquid chromatography (UHPLC) analysis using an Agilent Technologies 1290 Infinity UHPLC apparatus equipped with a diode array detection (DAD) and Shield RP C18-column (2.19 × 100 mm, 1.7 μm pore size) at a temperature of 35 °C. Details of the analysis were presented earlier [38]. The compounds were identified by comparing the retention times of peaks (Rt), UV, and mass spectra of the samples with those of standards. The identification procedure is described in detail by Weremczuk-Jeżyna et al. [22]. Standards of rosmarinic acid, chlorogenic acid, and apigenin-7-*O*-glucoside were purchased from Sigma-Aldrich (Taufkirchen, Germany). When a pure standard was unavailable, we quantified the identified compound against the calibration curve of a similar compound—acacetin and apigenin derivatives were quantified according to apigenin-7-*O*-glucoside, and dicaffeoylquinic acid according to caffeic acid. The regression equations were for caffeic acid y = 1820.528x (*r*^2^ = 0.999), for chlorogenic acid y = 970.279 (*r*^2^ = 0.999), for rosmarinic acid y = 980.968x (*r*^2^ = 0.999), and for apigenin-7-*O*-glucoside y = 149.724x (*r*^2^ = 0.999). The metabolite content was expressed in milligram per gram DW.

### 3.4. Biological Investigations

#### 3.4.1. Preparation of Samples 

The transformed shoots from the NSB were lyophilized and powered, and 1.0 g samples were extracted in methanol/water as described above for phytochemical analysis. The obtained dry hydromethanolic extract was used for biological investigations.

#### 3.4.2. Antioxidant Potential

The antioxidant potential of the extract from the NSB-grown *D. forrestii* shoots was determined using the ferric reducing antioxidant power (FRAP), 1,1-diphenyl-2-picrylhydrazyl radical (DPPH), and super anion radical (O_2_^•−^) assays. For the FRAP assay, we determined antioxidant activity spectrophotometrically against a calibration curve of ferrous sulfate according to Grzegorczyk-Karolak et al. [37]; the absorbance was measured at 595 nm and expressed as μM Fe(II) g/DW of extract. For the DPPH test, we determined radical scavenging activity according to Weremczuk-Jeżyna et al. [76]; absorbance was measured after 30 min at 517 nm, and antiradical activity was expressed as EC_50_ value (μg/mL), i.e., the concentration of the sample required to reduce initial DPPH concentration by 50%. Superoxide anion radical reduction was evaluated by the reduction of NBT according to Grzegorczyk-Karolak and Kiss [77]. The activity of the extract was measured at 560 nm and calculated as EC_50_, i.e., the concentration of the sample demonstrating 50% of maximum absorption (μg/mL). The synthetic antioxidant BHT (butylated hydroxytoluene) was used in all antioxidant assays as a control.

#### 3.4.3. Antimicrobial Potential

The antimicrobial test was performed using reference microorganisms from the American Type Culture Collection (ATCC) including the following: *Staphylococcus aureus* ATCC 6538, *Staphylococcus epidermidis* ATCC 12228, *Enterococcus faecalis* ATCC 29212, *Escherichia coli* ATCC 25922, *Pseudomonas aeruginosa* ATCC 27833, *Candida albicans* ATCC 10231, and *Aspergillus brasiliensis* ATCC 16404. The *Enterococcus faecium* PCM 1859 and *Bacillus cereus* PCM 1948 strains were taken from the Polish Collection of Microorganisms (PCM). The *Proteus vulgaris* CCM 1799 strain was obtained from the Czech Collection of Microorganisms. Two yeast-like fungal strains of *Candida albicans* ZMF 1 and *Candida glabrata* 2 ZMF were derived from the collection of the Department of Pharmaceutical Microbiology and Diagnostic Microbiology, Medical University of Lodz. All tested microorganisms were stored at −80 °C in 15% glycerol stocks. Before testing, the bacterial strains were transferred on Mueller–Hinton agar (Oxoid) and cultured overnight at 37 °C. Fungal strains were transferred on Sabouraud agar (Oxoid) and cultured for 2 days at 30 °C. 

The antimicrobial activity of the extracts was evaluated by determination of MIC (minimum inhibitory concentrations), i.e., the lowest concentration that completely inhibits the growth of microorganisms, and the MBC (minimum bactericidal concentrations), the lowest concentration of extract that kills > 99.9% of the initial microorganism population. Before the analysis, we dissolved dry plant extract in sterile 10% DMSO (Sigma-Aldrich, Darmstad Germany) to a stock solution 10 mg/mL. Investigation was performed using the broth microdilution method as described by Grzegorczyk-Karolak et al. [78]. Mueller–Hinton broth (pH ≈ 7.2) was used for bacteria. Liquid medium RPMI-1640 (Roswell Park Memorial Institute) (without phenol red) (pH ≈ 7.2) was used for the fungal strains. Twofold series dilutions of extract in the growth medium were performed in 96-well sterile microtiter plates (Kartell Labware, Noviglio, Italy) at concentrations ranging from 0.010 to 20 mg/mL. Rosmarinic acid, amikacine, and fluconazole (all from Sigma-Aldrich, Darmstad, Germany) were used as reference antimicrobials.

#### 3.4.4. Cytotoxicity Properties

The L929 mouse fibroblasts (LGC Standards, Middlesex, UK) were used for in vitro cytotoxicity testing. The cells were maintained under standard conditions (37 °C, 5 % CO_2_) in 25 mL tissue culture flasks in RPMI-1640 medium supplemented with 10% fetal bovine serum (FBS) and antibiotics: 100 U/mL penicillin and 100 μg/mL streptomycin. To obtain a cell suspension for the cytotoxicity assay, or for starting a new culture, we treated confluent monolayers with 0.25% trypsin solution, washed them, and subcultured them at a cell density of 10^8^ cells/mL. Cell cultures were supplemented with fresh medium 2 or 3 times per week to maintain them in log phase. The viability of the cells was assessed by exclusion of trypan blue dye and was in the range of 93–95%.

The hydromethanolic extract obtained from the transformed *D. forrestii* shoots was used to assess L929 cell metabolic activity. Cells in culture medium were seeded in the 96-well plates (2 × 10^5^ cells per well) for 24 h at 37 °C, 5% CO_2_. The tested extracts were diluted in RPMI-1640 medium at the following concentrations: 0.05, 0.075, 0.1, 0.25, 0.5, 0.75, 1.0, 2.5, 5.0, 10, 25, and 50 mg/mL. They were then added to the cells (100 μL/well) and incubated under standard conditions for 24 h. Following incubation, the cell monolayers were carefully screened using light microscopy, as recommended by ISO (International Organization for standardization) norm 10993-5 to evaluate the morphology of cells [79].

Cell viability was estimated on the basis of their ability to reduce MTT (3-(4,5-dimethylthiazol-2-yl)-2,5-diphenyltetrazolium bromide), as recommended by the Food and Drug Administration (FDA) and the International Organization for Standardization (ISO). Fresh MTT solution (5 mg /mL in sterile PBS (phosphate buffered saline)) was added to each well and incubated for 4 h at 37 °C. Formazan crystals were dissolved with acidic isopropanol (0.1 M HCl in absolute isopropanol). Optical density values were measured at 570 nm with a Victor2plate reader (Wallac Oy, Turku, Finland). The results are presented as the mean number of cells able to convert MTT obtained from 4 independent experiments, including 3 repeats for tested extracts. Complete RPMI medium (cRPMI) was used as a positive control (PC) of cell viability (100% viable cells) and 0.03% H_2_O_2_ as a negative control (NC) of cell viability (100% dead inactive cells).

### 3.5. Statistical Analysis

Data are presented as mean values ± standard error (SE). For statistical analysis, we used the STATISTICA 10 PL software (Statsoft, Kraków, Poland). Results were compared using the non-parametric Mann–Whitney *U* test. Statistical significance was accepted at a *p* value < 0.05. 

## 4. Conclusions

The scale-up of *D. forrestii* transformed shoot culture allowed us to obtain significant amounts of plant material rich in bioactive metabolites. The most favorable system for the development of culture was the NSB, in which biomass of shoots increased 40 times (to 42 g) after 3 weeks of growth. At the same period, 131.5 mg phenolic compounds—96.9 mg of RA, more than 20 mg acacetin derivatives, and around 10 mg apigenin derivatives—were produced. The NSB system shoot culture revealed moderate antioxidant and antimicrobial potential. In order to increase the therapeutic potential of *D. forrestii* transformed shoots, it is necessary to fractionate the extract and obtain a more concentrated product with respect to bioactive compounds. In addition, the lack of cytotoxic activities of the extract towards healthy cells indicates its safety.

## Figures and Tables

**Table 1:** The growth parameters of transformed shoot culture of *Dracocephalum forrestii* after 3 weeks in different bioreactor systems in MS (Murashige and Skoog) medium with 0.5 mg/L BPA and 0.2 mg/L IAA.

Type of Bioreactors	Number of Shoots per Explants	Number of Shoots per Bioreactor	Growth Index		Hyperhydricity Shoots (%)
			FW	DW	
RITA ^1^	47.6 ± 5.1	286 ± 10.2	52.06 ± 1.2	55.67 ± 4.6	16
Plantform ^2^	17.02 ± 3.1	204 ± 8.5	11.89 ± 0.4	8.65 ± 0.3	5
NSB^2^	69.7 ± 7.3	836 ± 26.9	43.48 ± 1.2	44.55 ± 0.7	24

The results are expressed as means of three replicates ± standard error (SE). ^1^ 5–7 explants (fragment shoots with nodes) in bioreactors; ^2^ 10–14 explants (fragment shoots with nodes) in bioreactor.

**Table 2:** Phenolic content in hydromethanolic extract of *D. forrestii* transformed shoots grown for 3 weeks in different bioreactor systems in MS medium with BPA 0.5 mg/L and IAA 0.2 mg/L.

Bioreactor Type	Content of Compounds	Number of Compounds							
		1	2 *	3	4	5 *	6 *	7	8
RITA	mg/g DW	0.99 ± 0.02 ^a^	tr	1.01 ± 0.03 ^a^	11.91 ± 0.1 ^b^	tr	tr	2.45 ± 0.02 ^a^	1.86 ± 0.01 ^b^
	mg/L	13.0 ± 1.3 ^A^	tr	13.28 ± 1.2 ^A^	156.6 ± 4.1 ^A^	tr	tr	32.2 ± 2.6 ^A^	24.44 ± 2.8 ^A^
Plantform	mg/g DW	0.78 ± 0.03 ^c^	tr	0.97 ± 0.05 ^a,b^	8.23 ± 0.1 ^c^	tr	tr	2.34 ± 0.04 ^a^	2.28 ± 0.03 ^a^
	mg/L	1.43 ± 0.2 ^C^	tr	1.74 ± 0.3 ^C^	29.64 ± 2.8 ^C^	tr	tr	4.22 ± 0.1 ^C^	4.11 ± 0.3 ^C^
NSB	mg/g DW	0.88 ± 0.01 ^b^	tr	0.91 ± 0.03 ^b^	18.35 ± 0.2 ^a^	tr	tr	2.25 ± 0.1 ^a^	1.76 ± 0.04 ^b^
	mg/L	4.09 ± 0.2 ^B^	tr	8.81 ± 0.1 ^B^	96.97 ± 2.7 ^B^	tr	tr	11.89 ± 0.4 ^B^	9.29 ± 0.5 ^B^

Compounds: (1) chlorogenic acid, (2) dicaffeoylquinic acid, (3) acacetin rhamnosyl-trihexoside, (4) rosmarinic acid, (5) apigenin caffeoylrhamnoside, (6) apigenin *p*-coumaroylrhamnoside (I), (7) acacetin acetylrhamnosyl-trihexoside, (8) apigenin *p*-coumaroylrhamnoside (II); * content of compound was less than 0.3 mg/g DW (tr: trace). The results are expressed as means of three replicates ± SE. Means followed by various letters (small letters for amount compounds in mg/g dry weight (DW) and big letters for amount of compounds in mg/bioreactor) within the columns were significantly different according to the Mann–Whitney *U* test (*p* < 0.05).

**Table 3:** Antioxidant capacity of hydromethanolic extract from transformed shoots of *D. forrestii* grown for 3 weeks in nutrient sprinkle bioreactor (NSB) in MS medium with BPA 0.5 mg/L and IAA 0.2 mg/L.

Assay	BHT	Plant Extract
FRAP (μM Fe(II)/g DW of extract)	3667.4 ± 52.0	724.9 ± 16.8
DPPH (EC_50_ μg/mL)	29.42 ± 0.1	74.9 ± 0.9
NBT (EC_50_ μg/mL)	42.7 ± 0.1	116.1 ± 9.0

The results are expressed as means of three replicates ± SE.

**Table 4:** Antimicrobial activity of hydromethanolic extract from *D. forrestii* transformed shoots cultured for 3 weeks in NSB in MS medium with BPA 0.5 mg/L and IAA 0.2 mg/L.

Microorganism	MIC mg/mL	MBC mg/mL	RA MIC/MBC mg/mL	Amikacine MIC = MBC µg/mL	Fluconazole MIC/MB µg/mL
**Gram-Positive Bacteria**					
*Staphylococcus aureus* ATCC 6538	>20	>20	1/>1	0.5	-
*Staphylococcus epidermidis* ATCC 12228	10	10	1/>1	0.125	-
*Enterococcus faecalis* ATTC 29212	>20	>20	1/>1	8	-
*Enterococcus faecium* PCM 1859	>20	>20	1/>1	2.5	-
*Bacillus cereus* PCM 1948	10	10	1/1	0.03	-
**Gram-Negative Bacteria**					
*Escherichia coli* ATCC 25922	10	10	1/1	0.625	-
*Pseudomonas aeruginosa* ATCC 27853	>20	>20	1/>1	20	-
*Proteus vulgaris* CCM 1799	>20	>20	1/1	0.625	-
**Fungi**					
*Candida albicans* ATTC 10231	>20	>20	1/>1	-	5/>5
*Candida albicans* ZMF 1	>20	>20	1/>1		5/>5
*Candida glabrata* 2 ZMF	>20	>20	0.5/0.5	-	2.5/>5
*Aspergillus brasilinsis* ATCC 16404	>20	>20	1/>1	-	5/>5

MIC: minimum inhibitory concentration; MBC: minimum bactericidal concentration. Amikacine broad-spectrum antibiotic and fluconazole broad-spectrum antifungal chemotherapeutic were employed as standard antimicrobials; (−): not tested.

## Supplementary Materials

The following are available online.
