# Supplementary material for: Transformed Shoots of Dracocephalum forrestii W.W. Smith from Different Bioreactor Systems as a Rich Source of Natural Phenolic Compounds
Source: Molecules. 2020 Oct 3;25(19):4533. doi: 10.3390/molecules25194533 (PMC7583972; doi:10.3390/molecules25194533)
Supplement: Supplementary file 1 [file molecules-25-04533-s001.pdf]

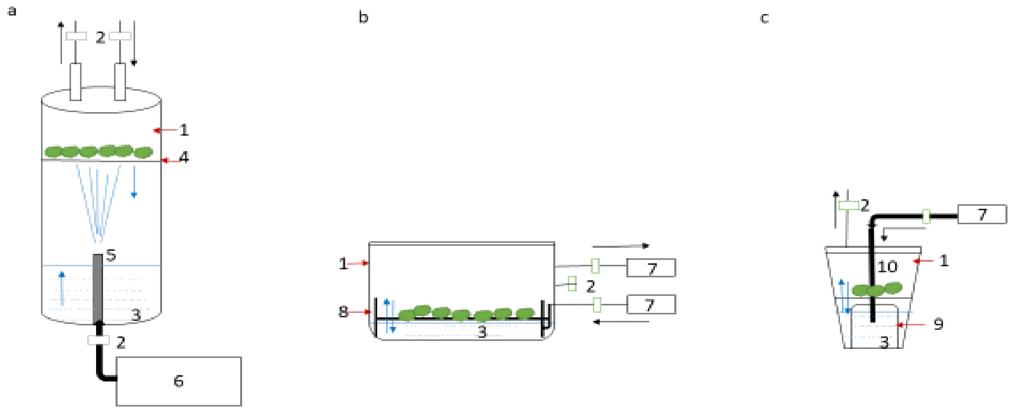

Figure S1. Diagrams of bioreactors: a) nutrient sprinkle bioreactor (NSB), b) Plantform bioreactor, c) RITA: 1-growth vessel, 2-air filter, 3-growth medium, 4-stainless stell, 5-spray nozzle, 6-peristaltic pump, 7- air pomp, 8-basket with 3 rows of small pors, 9- inner chamber, 10-compressed tube. Blue arrows indicate the flow direction of growth medium, black arrows indicate the flow direction of air.
